# Supplementary material for: Estimating the optimal linear combination of predictors using spherically constrained optimization
Source: BMC Bioinformatics. 2022 Oct 19;23(Suppl 3):436. doi: 10.1186/s12859-022-04953-y (PMC9583504; doi:10.1186/s12859-022-04953-y)
Supplement: Supplementary file 1 — Additional file 1. This file contains a brief description about the literature on existing global optimization techniques, existing techniques alternative to SCOR, theoretical properties and optimization performance evaluation of SCOR, additional simulation results, and an application of SCOR to Alzheimer's disease data. Table S1. Tuning parameters, their roles and default values in the SCOR algorithm. Table S2. Comparison of minimum value achieved and average computation time (in seconds) for solving the transformed d-dimensional negative log-product function, modified Griewank’s function, negative sum of squares function, modified Exponential function, and modified Easom function for d = 5, 20, 50, 100 and 500 using SCOR, PS, GA, SA starting from 10 randomly generated points in each case. Table S3. Performance comparison for two and three ordinal outcomes, where each class has sample size 60 for the two category case, and 30 for the three category case. The empirical hypervolume under manifolds (EHUM) and upper and lower bound approach (ULBA) objective functions are maximized by the proposed Spherically Constrained Optimization Routine (SCOR) algorithm and the existing Nelder-Mead (NM), step-down, and min-max algorithms. The estimated biomarker coefficient vectors are then used to calculate the EHUM value on a new dataset of the same size generated from the corresponding model. The entire procedure is repeated 100 times, resulting in 100 simulated training and test data sets, and the mean EHUM objective function values on the test data are reported, with the standard error in the parentheses. The result for the method with the best performance is marked in bold. [file 12859_2022_4953_MOESM1_ESM.pdf]

Supplementary Material for "Estimating the optimal linear combination of predictors using spherically constrained optimization"

Priyam Das
Department of Biomedical Informatics, Harvard Medical School
Debsurya De
Indian Statistical Institute
Raju Maiti
Centre for Quantitative Medicine,
Duke-National University of Singapore Medical School
Mona Kamal
Department of Radiation Oncology, University of Texas MD Anderson Cancer Center
Katherine A. Hutcheson
Department of Head and Neck Surgery, University of Texas MD Anderson Cancer Center
Clifton D. Fuller
Department of Radiation Oncology, University of Texas MD Anderson Cancer Center
Bibhas Chakraborty
Centre for Quantitative Medicine,
Duke-National University of Singapore Medical School
Department of Statistics and Applied Probability, National University of Singapore
Department of Biostatistics and Bioinformatics, Duke University
and Christine B. Peterson
Department of Biostatistics, University of Texas MD Anderson Cancer Center

March 9, 2022

Contents

A Global optimization **2**

B Existing techniques for estimating the optimal β **3**

B.1 The step-down algorithm 3

B.2 The min-max technique 4

C Modification for better convergence of SCOR 4

D Proof of theorems 6

E Performance of SCOR on benchmark functions 11

F Additional simulation results 15

G Application to Alzheimer's disease data 16

A Global optimization

In this section, we provide additional background on global optimization procedures. For maximizing any multi-modal function, global optimization techniques such as the genetic algorithm (GA; Fraser 1957, Bethke 1980) and simulated annealing (SA; Kirkpatrick et al. 1983, Granville et al. 1994) have been shown to yield better results compared to regular convex optimization methods such as the interior-point algorithm (IP; Karmakar 1984, Potra & Wright 2000) or the sequential quadratic programming algorithm (SQP; Wright 2005, P.T. Boggs 1996). In most global optimization techniques, unlike convex optimization algorithms, once a local maximum is reached, based on some heuristic principles, attempts are made to look for a possible better solution in a different neighbourhood. Global optimization techniques provide better results compared to convex optimization methods when dealing with multi-modal objective functions. However, for our problem of interest, with an increasing number of biomarkers, most existing global optimization techniques become computationally too expensive. Specifically for GA, there is an exponential increase in the search space with the dimension of the parameter space (Geris 2012). Due to the excessive time consumption of existing global optimization techniques, to maximize the estimates of HUM, only convex optimization techniques are generally used, with or without using the step-down principle.

In addition to GA and SA, over the last few decades, several deterministic and stochastic global search algorithms have been proposed over unconstrained and constrained parameter spaces (Nelder & Mead 1965, Steihaug & Suleiman 2013). In the direct search algorithm for unconstrained optimization, first introduced by Hooke & Jeeves (1961), in each iteration a set of possible solution points are chosen around the current solution without using gradient-based techniques. Then, the best solution point is chosen out of the combined set of possible solutions including the current solution and the new set of possible solutions. Extending the idea of direct search, Torczon (1997) proposed generalized pattern search (GPS), where the possible solution points around the current solution are found by moving along the coordinates with a step-size vector, derived using an algorithm of exploratory

moves. Later, Kolda et al. (2003) and Audet & Dennis (2006) further generalized GPS into the generating set search (GSS) and mesh adaptive direct search (MADS) algorithms, respectively. Although other global optimization tools have been proposed (see for example Audet et al. 2008, Conn et al. 2009, Digabel 2011), most of them deal with unconstrained optimization problems.

B Existing techniques for estimating the optimal $\boldsymbol{\beta}$

In this section, we review the most popular algorithms for estimating the optimal value of the combination vector $\boldsymbol{\beta}$.

B.1 The step-down algorithm

When maximizing a non-concave function, as the dimension of the parameter space increases, it becomes harder for any given algorithm to reach the true solution. In the step-down approach (Pepe et al. 2006), all the biomarkers are first ranked according to their importance. The coefficient of the first (i.e., the most important) biomarker is taken to be 1. Then at each step, one new biomarker is included and its coefficient is calculated without changing the coefficients of the other already included biomarkers. Thus, the problem of maximizing an objective function of an m-dimensional parameter is broken down into $d-1$ univariate maximization problems. As step-down is a strategy for maximizing any given objective function, any HUM estimate can be solved using this strategy. Because the performance of most optimization algorithms declines with the increasing dimension of the parameter space, the step-down strategy is commonly used (e.g., Maiti et al. 2019) when combining more than 2 biomarkers.

The step-down algorithm to maximize any given objective function $D(\cdot)$, for example, EHUM or ULBA, goes as follows:

Step 1. EHUM values are computed for the individual biomarkers, which are arranged in decreasing order by their EHUM values. $X_{(1)}$ and $X_{(d)}$ would therefore have the highest and the lowest individual EHUM values, respectively.

Step 2. The first two biomarkers with the highest EHUM values are taken and combined as $V_2 = X_{(1)} + \lambda_2 X_{(2)}$, where λ_2 is a parameter that needs to be estimated.

Step 3. $D(\cdot)$ for the combined marker V_2 is maximized with respect to λ_2. Let $\widehat{V}_2 = X_{(1)} + \widehat{\lambda}_2 X_{(2)}$ denote the updated combination vector.

Step 4. For $i = 3, \ldots, d$ define $V_i = \widehat{V}_{i-1} + \lambda_i X_{(i)}$ and maximize $D(\cdot)$ with respect to λ_i. The combination vector obtained at i-th step is given by $\widehat{\lambda}_i$.

The estimated optimal marker $\widehat{V}_d = X_{(1)} + \widehat{\lambda}_2 X_{(2)} + \cdots + \widehat{\lambda}_d X_{(d)}$ is obtained at the end of Step 4.

B.2 The min-max technique

Liu et al. (2011) proposed the min-max (MM) method in the context of binary outcome, where, corresponding to each vector of biomarkers of length d, only the maximum and the minimum values of those d values are used to estimate the combination coefficient vector. Suppose $\mathbf{X}_{j,i_j} = (X_{j,i_j,1}, \ldots, X_{j,i_j,d})$ and $X_{j,i_j,max} = \max_{1\le k\le d} X_{j,i_j,k}$ and $X_{j,i_j,min} = \min_{1\le k\le d} X_{j,i_j,k}$. Consider the linear combination of these two quantities as $V_{j,i_j} = \beta_{max} X_{j,i_j,max} + \beta_{min} X_{j,i_j,min}$, $i = 1, 2, \cdots, n_j$, $j = 1, 2, \cdots, M$. Then the objective function to be maximized using the EHUM and ULBA approaches are given by:

$$D_E^{(MM)}(\beta_{max}, \beta_{min}) = \frac{1}{\prod_{j=1}^{M} n_j} \sum_{i_1=1}^{n_1} \sum_{i_2=1}^{n_2} \cdots \sum_{i_M=1}^{n_M} I(V_{M,i_M} > V_{M-1,i_{M-1}} > \cdots > V_{1,i_1}),$$

$$P_A^{(MM)}(\beta_{max}, \beta_{min}) = \frac{1}{M-1} \sum_{j=1}^{M-1} P(V_{j+1} > V_j),$$

where $P(V_{j+1} > V_j) = \frac{1}{n_j n_{j+1}} \sum_{i_j=1}^{n_j} \sum_{i_{j+1}=1}^{n_{j+1}} I(V_{j+1,i_{j+1}} > V_{j,i_j})$. Irrespective of the dimension of the biomarker vector, here the number of parameters to be estimated is only 2. In order to avoid the non-identifiability issue, in practice β_{max} is taken to be 1 and only β_{min} is estimated (Hsu & Chen 2016, Maiti et al. 2019, Liu et al. 2011). The solutions obtained by maximizing the above-mentioned modified objective function for EHUM and ULBA are denoted by:

$$\hat{\boldsymbol{\beta}}_E^{(MM)} = \underset{(\beta_{max}, \beta_{min}) \in \mathrm{R}^2}{\arg\max} \; D_E^{(MM)}(\beta_{max}, \beta_{min}),$$

$$\hat{\boldsymbol{\beta}}_A^{(MM)} = \underset{(\beta_{max}, \beta_{min}) \in \mathrm{R}^2}{\arg\max} \; P_A^{(MM)}(\beta_{max}, \beta_{min}).$$

C Modification for better convergence of SCOR

A discussion on how to choose the adjustment step size for SCOR is provided in the methods section of the main paper. After solving equation (3) of the main paper, we obtain

two possible solutions for the corresponding adjusted step sizes which are $T_1(s)$ and $T_2(s)$ (provided in equation (4) of the main paper). As shown in Section D below, by considering only $T_1(s)$ as the adjusted step size, theoretical properties under the regularity conditions can be derived for SCOR. Although considering $T_2(s)$ as the adjustment step size along with $T_1(s)$ does not offer any improvement in a theoretical sense, by extensive experimental study we have observed that considering both $T_1(s)$ and $T_2(s)$ as the adjustment sizes generally results in faster convergence with better results. Corresponding to the step size s for any given position of the array, using both $T_1(s)$ and $T_2(s)$ as adjustment step sizes, we obtain 4 candidate points (instead of 2, which occurs when we only consider $T_1(s)$ as the adjusted step size). Thus, for any given step size s, in total, we get $4n$ candidate solutions (instead of $2n$). We employ this strategy for further improvement of SCOR in convergence and computation time in the simulation study and application included in the main paper and comparative performance over benchmark functions (Section E). An overview of the SCOR parameters is given in Table S1 below.

D Proof of theorems

Theorem 1 *Suppose* $\mathbf{S} = \{(x_1, \cdots, x_n) \in \mathrm{R}^n : \sum_{i=1}^n x_i^2 = 1, i = 1, \cdots, n\}$. *Consider a sequence of step sizes* $\delta_k = \frac{s}{\rho^k}$ *for* $k \in \mathrm{N}$ *and* $s \neq 0, \rho > 1$. *Then there exists a* K *such that for* $k \geq K$, *all adjustment step sizes* $\{t_i\}_{i=1}^d$ *are real.*

Proof: [Proof of Theorem 1] From Equation (4) of the main paper, the adjustment step size t_i as a function of δ_k is given by

$$t_i(\delta_k) = \frac{-2\sum_{k=1,k\neq i}^n \beta_k^{(j)} + \sqrt{D_i(\delta_k)}}{2(n-1)}, i = 1, \ldots, n,$$

$$D_i(\delta_k) = \big(2\sum_{k=1,k\neq i}^n \beta_k^{(j)}\big)^2 - 4(n-1)(2\delta_k\beta_i^{(j)} + \delta_k^2).$$

Note that $\delta_k \to 0$ as $k \to \infty$. Hence,

$$\lim_{k\to\infty} D_i(\delta_k) = \big(2\sum_{k=1,k\neq i}^n \beta_k^{(j)}\big)^2$$

Since $D_i(\delta_k)$ is a continuous function of δ_k, if we take k to be sufficiently large, we can make $D_i(\delta_k) \geq 0$. Suppose for $k \geq K_i$, $D_i(\delta_k) \geq 0$ holds true for $i = 1, \ldots, n$. Take $K = \max_{1\leq i\leq n} K_i$, hence, for all $k \geq K$, t_i is real for $i = 1, \ldots, n$. □

Parameter	Description	Role	Recommended values and comments
$s_{initial}$	initial global step size	Initial step size at the beginning of the run, higher value promotes selection of distant candidate solutions.	2 (setting it 2 allows maximum possible coordinate-wise jump on unit-sphere space)
ρ	step decay rate	Controls the rate of decay of global step-size, smaller value of ρ results in slower decay of the global step size, thus it allows denser search in the neighborhood of the current solution at the expense of higher computation time.	2 (must be > 1)
ϕ	lower bound of global step size	Controls precision of search, smaller value of ϕ results in more accurate solution in the expense of higher computation time.	10^{-20}
λ	sparsity threshold	(i) Controls sparsity, encourage sparse solution. (ii) Helps in the search procedure when coordinate(s) of the starting point of any iteration is(are) close to 0.	10^{-6} (may consider 10^{-2} or 10^{-1} for inducing more sparsity, or can be set as small as 0)
tol_fun	termination tolerance on the function value	The minimum amount of improvement in objective function value required so that the global step size is not reduced after the iteration.	10^{-6}
tol_fun_2	termination tolerance on the difference of solutions of two consecutive *runs*	The minimum euclidean distance between solutions of two consecutive *runs* so that next *run* is executed.	10^{-20}
max_runs	max no. of runs	Put an upper limit on number of runs.	1000 (however the algorithm converged before 1000 runs in all the cases considered in this article)
max_iter	max no. of iterations	Put an upper limit on number of iterations allowed within each run.	10000 (however required number of iterations within a run never crossed 10000 in any of the considered cases)

Table S1: Tuning parameters, their roles and default values in the SCOR algorithm.

Proposition 1 *Consider a matrix* $\mathbf{A} = (a_{ij})_{(n-1)\times(n-1)}$ *such that* $a_{ii} = 1$ *for* $i = 1, \ldots, n-1$ *and* $a_{ij} = b_i$ *for* $i \neq j, i = 1, \ldots, n-1, j = 1, \ldots, n-1$. *Then* A *is full rank for* $n \in \mathbb{N} \setminus \{1\}$ *iff*

1. $1 - b_i \neq 0$ *for* $i = 1, \ldots, n-1$.
2. $\left[(n-2) + \sum_{i=1}^{n-1} \frac{1}{1-b_i}\right] \neq 0$.

Proof: We have

$$\mathbf{A} = \begin{bmatrix} 1 & b_1 & \cdots & b_1 \\ b_2 & 1 & \cdots & a_2 \\ \vdots & \vdots & \ddots & \vdots \\ b_{(n-1)} & b_{(n-1)} & \cdots & 1 \end{bmatrix}.$$

By performing a series of column operations $C_i : C_i - C_{n-1}$ for $i = 1, \ldots, n-2$, we obtain $\mathbf{A}'$ as follows:

$$\mathbf{A}' = \begin{bmatrix} 1-b_1 & 0 & \cdots & b_1 \\ 0 & 1-b_2 & \cdots & b_2 \\ \vdots & \vdots & \ddots & \vdots \\ b_{(n-1)}-1 & b_{(n-1)}-1 & \cdots & 1 \end{bmatrix}$$

Now consider the following series of row and column operations

$$\begin{bmatrix} 1-b_1 & 0 & \cdots & b_1 \\ 0 & 1-b_2 & \cdots & b_2 \\ \vdots & \vdots & \ddots & \vdots \\ b_{(n-1)}-1 & b_{(n-1)}-1 & \cdots & 1 \end{bmatrix} \xrightarrow{C_{n-1}:C_{n-1}+\sum_{i=1}^{n-2} C_i}$$

$$\begin{bmatrix} 1-b_1 & 0 & \cdots & 1 \\ 0 & 1-b_2 & \cdots & 1 \\ \vdots & \vdots & \ddots & \vdots \\ b_{(n-1)}-1 & b_{(n-1)}-1 & \cdots & (n-2)(b_{(n-1)}-1)+1 \end{bmatrix} \xrightarrow{R_{n-1}:R_{n-1}/(b_{(n-1)}-1)}$$

$$\begin{bmatrix} 1-b_1 & 0 & \cdots & 1 \\ 0 & 1-b_2 & \cdots & 1 \\ \vdots & \vdots & \ddots & \vdots \\ 1 & 1 & \cdots & (n-2)+\frac{1}{b_{(n-1)}-1} \end{bmatrix} \xrightarrow{C_{n-1}:C_{n-1}+\sum_{i=1}^{n-2} \frac{1}{b_i-1} C_i}$$

$$\begin{bmatrix} 1-b_1 & 0 & \cdots & 0 \\ 0 & 1-b_2 & \cdots & 0 \\ \vdots & \vdots & \ddots & \vdots \\ 1 & 1 & \cdots & (n-2)+\sum_{i=1}^{n-1} \frac{1}{b_i-1} \end{bmatrix} \xrightarrow{R_{n-1}:R_{n-1}+\sum_{i=1}^{n-2} \frac{1}{b_i-1} R_i}$$

$$\begin{bmatrix} 1-b_1 & 0 & \cdots & 0 \\ 0 & 1-b_2 & \cdots & 0 \\ \vdots & \vdots & \ddots & \vdots \\ 0 & 0 & \cdots & (n-2)+\sum_{i=1}^{n-1} \frac{1}{b_i-1} \end{bmatrix}, \text{ which we denote as } \mathbf{A}''.$$

Since $\mathbf{A}''$ is diagonal matrix, the determinant of $\mathbf{A}''$ is given by

$$det(\mathbf{A}'') = \big[(n-2) + \sum_{i=1}^{n-1} \frac{1}{b_i - 1}\big] \prod_{i=1}^{n-2} (1 - b_i)$$

Clearly $r(\mathbf{A}) = r(\mathbf{A}'')$ where $r(\mathbf{B})$ denotes the rank of any given matrix $\mathbf{B}$. Hence $\mathbf{A}$ is full rank iff

1. $(1-b_i) \neq 0$ for $i = 1, \ldots, n-1$,

2. $\left[(n-2) + \sum_{i=1}^{n-1} \frac{1}{b_i - 1}\right] \neq 0$.

$\square$

Theorem 2 *Suppose* $\mathbf{S} = \{(x_1, \cdots, x_n) \in \mathrm{R}^n : \sum_{i=1}^{n} x_i^2 = 1, i = 1, \cdots, n\}$ *and* f *is convex, continuous and differentiable on* $\mathbf{S}$. *Consider a sequence* $\delta_k = \frac{s}{\rho^k}$ *for* $k \in \mathrm{N}$ *and* $s > 0, \rho > 1$. *Suppose* $\mathbf{u}$ *is a point in* $\mathbf{S}$. *Define* $\mathbf{u}_k^{(i+)} = (u_1 + t_i(\delta_k), \ldots, u_{i-1} + t_i(\delta_k), u_i + \delta_k, u_{i+1} + t_i(\delta_k), \ldots, u_n + t_i(\delta_k))$ *and* $\mathbf{u}_k^{(i-)} = (u_1 + t_i(-\delta_k), \ldots, u_{i-1} + t_i(-\delta_k), u_i - \delta_k, u_{i+1} + t_i(-\delta_k), \ldots, u_n + t_i(-\delta_k))$ *for* $i = 1, \cdots, n$, *where* $t_i(s)$ *denotes the adjustment step size corresponding to step size* s. *Define* $b_i = -\frac{u_i}{\left|\sum_{k=1, k\neq i}^{n} u_k\right|}$. *If the following conditions hold true*

1. $1 - b_i \neq 0$ *for* $i = 1, \ldots, n-1$,

2. $\left[(n-2) + \sum_{i=1}^{n-1} \frac{1}{1-b_i}\right] \neq 0$,

3. *for all sufficiently large* $k \in \mathrm{N}$, $f(\mathbf{u}) \leq f(\mathbf{u}_k^{(i+)})$ *and* $f(\mathbf{u}) \leq f(\mathbf{u}_k^{(i-)})$ *for all* $i = 1, \cdots, n$,

then the global minimum of f *over* $\mathbf{S}$ *occurs at* $\mathbf{u}$.

Proof: [Proof of Theorem 2] From Theorem 1, there exists a $K_1 \in \mathrm{N}$ such that for all $k \geq K_1$, $t_i(\delta_k)$ is real for $i = 1, \ldots, n$. Similarly it can be shown that there exists a $K_2 \in \mathrm{N}$ such that for all $k \geq K_2$, $t_i(-\delta_k)$ is real for $i = 1, \ldots, n$. Take $K = \max(K_1, K_2)$. Hence for all $k \geq K$, both $t_i(\delta_k)$ and $t_i(-\delta_k)$ are real for $i = 1, \ldots, n$, and, therefore, for $k \geq K$, $\mathbf{u}_k^{(i+)}$ and $\mathbf{u}_k^{(i-)} \in \mathbf{S}$ for all $i = 1, \ldots, n$. For the rest of the proof, we only consider the cases for $k \geq K$. Define

$$\mathbf{S}^+ = \{(x_1, \cdots, x_n) \in \mathrm{R}^n : \sum_{i=1}^{n} x_i^2 = 1, x_n \geq 0, i = 1, \cdots, n\},$$

$$\mathbf{S}^- = \{(x_1, \cdots, x_n) \in \mathrm{R}^n : \sum_{i=1}^{n} x_i^2 = 1, x_n < 0, i = 1, \cdots, n\}.$$

Note that $\mathbf{S} = \mathbf{S}^+ \cup \mathbf{S}^-$. So if we can prove this theorem on both $\mathbf{S}^+$ and $\mathbf{S}^-$, that would suffice. Suppose $(u_1, \ldots, u_n) \in \mathbf{S}^+$. The n-th coordinate u_n can be derived as a unique function of first $n-1$ coordinates given by $u_n = \sqrt{1 - \sum_{i=1}^{n-1} u_i^2}$. Define

$$\mathbf{S}^* = \{(x_1, \cdots, x_{n-1}) \in \mathrm{R}^{n-1} : \sum_{i=1}^{n} x_i^2 < 1, i = 1, \cdots, n-1\},$$

$$
\begin{aligned}
&\mathbf{u}^* = (u_1, \ldots, u_{n-1}),\\
&\mathbf{u}_k^{*(i+)} = (u_1 + t_i(\delta_k), \ldots, u_{i-1} + t_i(\delta_k), u_i + \delta_k, u_{i+1} + t_i(\delta_k), \ldots, u_{n-1} + t_i(\delta_k)),\\
&\mathbf{u}_k^{*(i-)} = (u_1 + t_i(-\delta_k), \ldots, u_{i-1} + t_i(-\delta_k), u_i - \delta_k, u_{i+1} + t_i(-\delta_k), \ldots, u_{n-1} + +t_i(-\delta_k)),
\end{aligned}
$$

for $i = 1, \ldots, n-1$. Note that $\mathbf{u}^*, \mathbf{u}_k^{*(i+)}$, and $\mathbf{u}_k^{*(i-)}$ are the first $(n-1)$ coordinates of $\mathbf{u}, \mathbf{u}_k^{(i+)}$, and $\mathbf{u}_k^{(i-)}$, respectively. Define $f^* : \mathbf{S}^* \mapsto \mathbb{R}$ such that

$$f^*(x_1, \ldots, x_{n-1}) = f(x_1, \ldots, x_{n-1}, \sqrt{1 - \sum_{i=1}^{n-1} x_i^2}).$$

Hence we have $f^*(\mathbf{u}^*) = f(\mathbf{u})$, $f^*({\mathbf{u}^*}_k^{(i+)}) = f(\mathbf{u}_k^{(i+)})$ and $f^*({\mathbf{u}^*}_k^{(i-)}) = f(\mathbf{u}_k^{(i-)})$. f is continuous and differentiable on $\mathbf{S}$, hence f is continuous and differentiable on $\mathbf{S}^+$. Therefore, f^* is continuous and differentiable on $\mathbf{S}^*$. Since f is convex on $\mathbf{S}$, f is also convex on $\mathbf{S}^+$.

We claim that f^* is convex on $\mathbf{S}^*$. Consider $\mathbf{x}_1^*, \mathbf{x}_2^* \in \mathbf{S}^*$. Suppose $\mathbf{x}_1, \mathbf{x}_2 \in \mathbf{S}^+$ are such that their first $(n-1)$ coordinates are the same as $\mathbf{x}_1^*$ and $\mathbf{x}_2^*$, respectively. Take any $\gamma \in (0, 1)$. Now

$$
\begin{aligned}
\gamma f^*(\mathbf{x}_1^*) + (1-\gamma) f^*(\mathbf{x}_2^*) &= \gamma f(\mathbf{x_1}) + (1-\gamma) f(\mathbf{x_2})\\
&\geq f(\gamma \mathbf{x}_1 + (1-\gamma)\mathbf{x}_2)\\
&= f^*(\gamma \mathbf{x}_1^* + (1-\gamma)\mathbf{x}_2^*).
\end{aligned}
$$

Hence f^* is also convex.
Define $h_i : U_i \mapsto \mathbf{S}^*$ such that

$$h_i(z) = (u_1 + t_i(z), \ldots, u_{i-1} + t_i(z), u_i + z, u_{i+1} + t_i(z), \ldots u_{n-1} + t_i(z))$$

for $i = 1, \ldots, n-1$, where $U_i = [-\delta_K, \delta_K]$. Note that $h_i(U_i) \subset \mathbf{S}^*$. Define $g_i : U_i \mapsto \mathrm{R}$ for $i = 1, \ldots, n-1$ such that $g_i = f^* \circ h_i$. Hence,

$$g_i(z) = f^*(u_1 + t_i(z), \ldots, u_{i-1} + t_i(z), u_i + z, u_{i+1} + t_i(z), \ldots u_{n-1} + t_i(z))$$

for $i = 1, \ldots, n-1$.

Note that h_i is continuous on $U_i = [-\delta_K, \delta_K]$ and differentiable on $(-\delta_K, \delta_K)$ for $i = 1, \ldots, n-1$. Also f^* is continuous and differentiable on $\mathbf{S}^*$. The composition of any two continuous functions is continuous. Also the composition of two differentiable functions is differentiable. Therefore, g_i is continuous on $U_i = [-\delta_K, \delta_K]$ and differentiable on $(-\delta_K, \delta_K)$.

For any $i \in \{1, \ldots, n-1\}$, $g_i(\delta_K) = f^*(\mathbf{u}_K^{*(i+)})$, $g_i(-\delta_K) = f^*(\mathbf{u}_K^{*(i-)})$ and $g_i(0) = f^*(\mathbf{u}^*)$. From the conditions provided in the theorem, we have $g_i(0) \leq g_i(-\delta_K)$ and $g_i(0) \leq g_i(\delta_K)$. Without loss of generality, suppose $f^*(\mathbf{u}_K^{*(i-)}) \leq f^*(\mathbf{u}_K^{*(i+)})$ which implies $g_i(0) \leq g_i(-\delta_K) \leq g_i(\delta_K)$.

Since $g_i(0) \leq g_i(-\delta_K) \leq g_i(\delta_K)$, from the continuity of g_i it can be said that there exists a $w \in [0, \delta_K]$ such that $g_i(w) = g_i(-\delta_K) \geq g_i(0)$. Since g_i is continuous on $[-\delta_K, \delta_K]$ and differentiable on $(-\delta_K, \delta_K)$, g_i is also continuous on $[-\delta_K, w]$ and differentiable on $(-\delta_K, w)$. Using the mean value theorem, there exists a point $v \in [-\delta_K, w]$ such that $g_i'(v) = 0$.

We claim that $g_i'(v) = 0$ holds for $v = 0$. Suppose $g_i'(0) \neq 0$ and $g_i'(v^*) = 0$ for some $v^* \in (-\delta_N, w) \setminus \{0\}$. Without loss of generality, take $v^* > 0$. Since h_i and f^* are convex on U_i and $\mathbf{S}^*$ respectively, g_i is also convex on $(-\delta_K, w) \subset U_i$. Now $g_i'(v^*) = 0$ implies v^* is a local minimum. Also $g_i'(0) \neq 0$, implies 0 is not a local minimum (or critical point). Therefore, $g_i(0) > g_i(v^*)$. Take $M \in \mathbb{N}$ such that it satisfies $0 < \delta_M < v^*$. Clearly, $K < M$ since $\delta_M < v^* \leq \delta_K$. Hence there exists a $\lambda \in (0, 1)$ such that $\delta_M = (1 - \lambda).v^* + \lambda.0$. So,

$$\begin{aligned} g_i(\delta_M) &= g_i((1-\lambda).v^* + \lambda.0) \\ &\leq (1-\lambda)g_i(v^*) + \lambda g_i(0) \\ &= g_i(0) - (1-\lambda)(g_i(0) - g_i(v^*)) \\ &< g_i(0). \end{aligned}$$

But, for all $k \geq K$, $g_i(0) \leq g_i(\delta_k)$, which implies $g_i(0) \leq g_i(\delta_M)$ (since $K < M$). It is a contradiction. Thus, $g_i'(0) = 0$. Now

$$\begin{aligned} g_i'(0) &= \left[\frac{\partial}{\partial \epsilon} g_i(\epsilon)\right]_{\epsilon=0} \\ &= \left[\frac{\partial}{\partial \epsilon} f^*(h_i(\epsilon))\right]_{\epsilon=0} \\ &= \left[\frac{\partial}{\partial h_i(\epsilon)} f^*(h_i(\epsilon))\right]_{\epsilon=0} \left[\frac{\partial}{\partial \epsilon} h_i(\epsilon)\right]_{\epsilon=0}. \end{aligned}$$

Now $h_i(0) = \mathbf{u}^*$. Hence

$$\begin{aligned} \left[\frac{\partial}{\partial h_i(\epsilon)} f^*(h_i(\epsilon))\right]_{\epsilon=0} &= \nabla f^*(\mathbf{u}^*) \\ &= \left[\frac{\partial}{\partial x_1} f^*(\mathbf{u}^*), \ldots, \frac{\partial}{\partial x_{n-1}} f^*(\mathbf{u}^*)\right] \\ &= \left[\nabla_1, \ldots, \nabla_{n-1}\right] \end{aligned}$$

where $\nabla_i = \frac{\partial}{\partial x_i} f^*(\mathbf{u}^*)$ for $i = 1, \ldots, n-1$.

$$[\frac{\partial}{\partial \epsilon} h_i(\epsilon)]_{\epsilon=0} = [a_{i1}, \ldots, a_{i(n-1)}]^T$$

where $a_{ii} = 1$ and

$$a_{ij} = \left.\frac{\partial t_i(s)}{\partial s}\right|_{s=o} = \frac{1}{2} \frac{-8(n-1)u_i}{2(n-1)\sqrt{(2\sum_{k=1,k\neq i}^{n} u_k)^2}} = -\frac{u_i}{|\sum_{k=1,k\neq i}^{n} u_k|} = b_i,$$

for $j \in \{1, \ldots, n-1\} \setminus \{i\}$. Hence

$$\begin{aligned} g_i'(0) = \left[\frac{\partial}{\partial \epsilon} g_i(\epsilon)\right]_{\epsilon=0} &= \Big[\nabla_1, \ldots, \nabla_{n-1}\Big]\Big[a_{i1}, \ldots, a_{i(n-1)}\Big]^T \\ &= \Big[a_{i1}, \ldots, a_{i(n-1)}\Big]\begin{bmatrix} \nabla_1 \\ \vdots \\ \nabla_{n-1} \end{bmatrix} \\ &= 0. \end{aligned}$$

Since this equation holds for all $i = 1, \cdots, n-1$, we have $\mathbf{Ax} = \mathbf{0}$ where

$$\mathbf{A}_{(n-1)\times(n-1)} = \begin{bmatrix} 1 & b_1 & \cdots & b_1 \\ b_2 & 1 & \cdots & b_2 \\ \vdots & \vdots & \ddots & \vdots \\ b_{n-1} & b_{n-1} & \cdots & 1 \end{bmatrix}, \ \mathbf{x}_{(n-1)\times 1} = \begin{bmatrix} \nabla_1 \\ \vdots \\ \nabla_{n-1} \end{bmatrix}.$$

By Proposition 1, $\mathbf{A}_{(n-1)\times(n-1)}$ is full-rank. Since $\mathbf{A}$ is full rank for $n \in \mathbb{N} \setminus \{1\}$, $\mathbf{Ax} = \mathbf{0}$ implies $\mathbf{x} = \mathbf{0}$. Hence $\frac{\partial}{\partial x_i} f^*(\mathbf{u}^*) = 0$ for all $i = 1, \ldots, n-1$. Hence $\mathbf{u}^*$ is a critical point. Since f^* is convex on $\mathbf{S}^*$, a local minimum occurs at $\mathbf{u}^*$. But for a convex function, the global minimum occurs at any local minimum. Hence the global minimum of f^* occurs at $\mathbf{u}^*$, which clearly implies the global minimum of f on $\mathbf{S}^+$ occurs at $\mathbf{u}$. Now, in case $\mathbf{u} \in \mathbf{S}^-$, similarly it can be shown that the global minimum of f on $\mathbf{S}^-$ occurs at $\mathbf{u}$. Hence the global minimum of f occurs at $\mathbf{u}$ for $\mathbf{u} \in \mathbf{S}$. □

E Performance of SCOR on benchmark functions

We compare the performance of SCOR with existing black-box optimization algorithms, namely the genetic algorithm (GA), simulated annealing (SA), and pattern search (PS), which were introduced in Section A above. The GA, SA and PS algorithms are available in the MATLAB 2016b toolbox under the functions `ga`, `simulannealbnd`, and `patternsearch`, respectively. Note that `ga` and `patternsearch` can be used to minimize any function whose parameters are on a unit sphere. However, the `simulannealbnd` function in MATLAB 2016b cannot handle non-linear constraints (to the best of our knowledge). Therefore while minimizing the following functions using simulated annealing, we minimize them over the compact region $[-1, 1]^d$ where d denotes the number of parameter coordinates in the objective functions. We use the default function parameter options for all of these aforementioned algorithms in MATLAB. SCOR is also coded in MATLAB 2016b. We use the

default parameter values as mentioned in Table S1. We consider the following benchmark functions on the parameter space $\mathbf{S}$ where

$$\mathbf{S} = \{(x_1, \ldots, x_d) : \sum_{i=1}^{d} x_i^2 = 1\}.$$

The explicit form of the following functions can be also found in Jamil & Yang (2013).

Example 1 (Negative log of product of absolute values):

$$f(x_1, \ldots, x_d) = -\sum_{i=1}^{d} \log |x_i| - \frac{d}{2} \log d.$$

The global minimum value is 0 which is attained at $(x_1, \ldots, x_d) = (\pm\frac{1}{\sqrt{d}}, \ldots, \pm\frac{1}{\sqrt{d}})$.

Example 2 (Modified Griewank function) :

$$f(x_1, \ldots, x_d) = \frac{1}{4000} \sum_{i=1}^{d} (x_i - \frac{1}{\sqrt{d}})^2 - \prod_{i=1}^{d} \cos \left[\frac{x_i - \frac{1}{\sqrt{d}}}{\sqrt{i}} \right] + 1.$$

The global minimum value is 0 which is attained at $(x_1, \ldots, x_d) = (\frac{1}{\sqrt{d}}, \ldots, \frac{1}{\sqrt{d}})$.

Example 3 (Negative sum of squares function):

$$f(x_1, \ldots, x_d) = d - \sum_{i=1}^{d} i x_i^2.$$

The global minimum value is 0 which is attained at $(x_1, \ldots, x_d) = (0, \ldots, 0, \pm 1)$.

Example 4 (Modified exponential function):

$$f(x_1, \ldots, x_d) = 1 - \exp(-0.5 \sum_{i=1}^{d-1} x_i^2).$$

The global minimum value is 0 which is attained at $(x_1, \ldots, x_d) = (0, \ldots, 0, \pm 1)$.

Example 5 (Modified Easom function):

$$f(x_1, \ldots, x_d) = 1 - \prod_{i=1}^{2} \cos(\sqrt{2}\pi x_i) \exp[-\sum_{i=1}^{2} (x_i - \frac{1}{\sqrt{2}})^2].$$

The global minimum value is 0 which is attained at $(x_1, \ldots, x_d) = (\frac{1}{\sqrt{2}}, \frac{1}{\sqrt{2}}, 0, \ldots, 0)$.

We minimize these functions for $d = 5, 20, 50, 100$, and 500 using the proposed SCOR algorithm and the existing algorithms GA, SA and PS. As shown in Table S2, SCOR generally outperforms other methods both in terms of the quality of the solution as well as computation times. Among other methods, PS performs better than GA or SA. For the high-dimensional modified Easom function, SA provides the best solution at the expense of a huge computational time. SCOR outperforms other methods in most of the scenarios. Using SCOR, we obtain up to a 67 fold improvement over PS in computation time (for the modified Griewank function for $d = 5$), up to a 43 fold improvement over SA (for the modified Easom function for $d = 20$) and up to a 38 fold improvement over GA (for the exponential function for $d = 5$).

Functions	Algorithms	$d = 5$		$d = 20$		$d = 50$		$d = 100$		$d = 500$	
		Min. value	Avg. time	Min. value	Avg. time	Min. value	Avg. time	Min. value	Avg. time	Min. value	Avg. time
Negative log-product function	SCOR	**1.78e - 15**	**2.66**	**9.24e - 14**	**77.72**	**8.81e - 13**	200.92	**8.38e - 12**	**646.82**	**1.45e - 08**	**3241.2**
	PS	**8.88e - 16**	10.67	**1.81e - 13**	415.56	**4.41e - 07**	4090.3	**2.66e - 05**	4123.6	**2.77e - 02**	3854.3
	GA	2.28e - 01	13.70	6.08e - 00	17.756	3.45e + 01	24.87	6.09e + 01	50.72	3.14e + 02	304.64
	SA	9.62e - 05	8.11	1.23e - 04	24.15	1.72e - 01	87.86	3.71e - 00	159.37	1.17e + 02	1630.0
Modified Griewank function	SCOR	**<1.00e - 16**	**0.45**	**<1.00e - 16**	**4.91**	**<1.00e - 16**	**28.59**	**1.11e - 16**	**132.98**	**1.20e - 11**	**3600**
	PS	**1.11e - 16**	30.43	**<1.00e - 16**	22.69	**8.88e - 16**	154.29	**1.11e - 16**	495.89	**1.13e - 04**	3869.8
	GA	1.72e -02	11.51	7.01e - 02	17.88	3.52e - 02	23.99	2.91e - 02	10.43	1.08e - 02	9.9688
	SA	9.11e -04	6.82	4.98e - 02	26.22	4.92e - 02	42.092	3.11e - 02	86.07	1.08e - 02	875.15
Negative Sum Squares function	SCOR	**<1.00e - 16**	**0.26**	**<1.00e - 16**	**6.35**	<1.00e - 16	**125.51**	**<1.00e - 16**	**442.28**	**<1.00e - 16**	**459.23**
	PS	**<1.00e - 16**	7.32	**<1.00e - 16**	3413.6	2.07e - 01	4086.6	**2.02e - 01**	4097.0	**5.27e - 01**	3882.8
	GA	4.63e - 01	9.54	3.90e - 00	13.59	1.32e + 01	24.13	3.03e + 01	59.926	6.67e + 01	347.73
	SA	5.43e - 04	7.10	4.81e - 01	30.67	2.74e + 01	73.27	1.89e + 02	477.85	1.56e + 04	3945.2
Modified Exponential function	SCOR	**<1.00e - 16**	**0.30**	**<1.00e - 16**	**1.81**	**<1.00e - 16**	**10.02**	**<1.00e - 16**	**41.98**	**<1.00e - 16**	**1503.9**
	PS	**<1.00e - 16**	1.56	**<1.00e - 16**	16.08	**1.11e - 16**	61.09	**2.22e - 16**	170.09	**1.11e - 15**	2455.5
	GA	1.41e - 01	11.45	2.93e - 01	22.17	3.70e - 01	18.19	3.89e - 01	16.58	3.92e - 02	11.772
	SA	4.29e - 04	8.20	3.06e - 01	16.93	3.70e - 01	42.75	3.89e - 01	86.32	3.92e - 02	715.66
Modified Easom function	SCOR	**<1.00e - 16**	**0.27**	**4.44e - 16**	**0.70**	**5.97e - 01**	**0.78**	**5.97e - 01**	**1.37**	**5.97e - 01**	**10.98**
	PS	**<1.00e - 16**	3.65	**<1.00e - 16**	4.49	**5.97e - 01**	6.74	**5.97e - 01**	12.90	**5.97e - 01**	68.37
	GA	7.35e - 03	5.68	6.08e - 01	16.74	6.09e - 01	14.66	6.76e - 01	10.35	6.24e - 01	8.00
	SA	1.11e - 05	7.39	2.75e - 06	30.32	**5.42e - 07**	82.02	**1.72e - 07**	156.96	**1.18e - 08**	1708.1

Table S2: Comparison of minimum value achieved and average computation time (in seconds) for solving the transformed d-dimensional negative log-product function, mdified Griewank's function, negative sum of squares function, modified Exponential function, and modified Easom function for $d = 5, 20, 50, 100$ and 500 using SCOR, PS, GA, SA starting from 10 randomly generated points in each case.

F Additional simulation results

In the results section of the main paper, we show the comparative performance of SCOR and other existing algorithms (NM, step-down and min-max) for two and three category outcome classification problems based on the existing objective functions estimating the hypervolume under manifolds (namely, ULBA and EHUM) with sample size 15 for each class. Here we provide an extended simulation study with sample sizes of 60 in each class for two category classification and with sample size 30 in each class for three category classification (in Table S3).

Number of categories and Sample sizes	d	Method	Scenario 1		Scenario 2		Scenario 3	
			ULBA	EHUM	ULBA	EHUM	ULBA	EHUM
$M=2$, $N=(60,60)$	5	SCOR	**0.958 (0.02)**	**0.958 (0.02)**	**0.984 (0.05)**	**0.984 (0.05)**	**0.939 (0.02)**	**0.939 (0.02)**
		NM	0.942 (0.03)	0.942 (0.03)	0.985 (0.02)	0.985 (0.02)	0.841 (0.08)	0.841 (0.08)
		Step-Down	0.524 (0.28)	0.524 (0.28)	0.503 (0.33)	0.503 (0.33)	0.933 (0.03)	0.933 (0.03)
		Min-Max	0.850 (0.05)	0.850 (0.05)	0.938 (0.02)	0.938 (0.02)	0.905 (0.03)	0.905 (0.03)
	10	SCOR	**0.995 (0.01)**	**0.995 (0.01)**	**0.998 (0.00)**	**0.998 (0.00)**	**0.997 (0.00)**	**0.997 (0.00)**
		NM	0.988 (0.01)	0.988 (0.01)	0.994 (0.01)	0.994 (0.01)	0.923 (0.05)	0.923 (0.05)
		Step-Down	0.471 (0.34)	0.471 (0.34)	0.442 (0.35)	0.442 (0.35)	0.995 (0.01)	0.995 (0.01)
		Min-Max	0.962 (0.02)	0.962 (0.02)	0.984 (0.01)	0.984 (0.01)	0.990 (0.01)	0.990 (0.01)
	15	SCOR	**0.997 (0.01)**	**0.997 (0.01)**	**0.997 (0.00)**	**0.997 (0.00)**	0.995 (0.01)	0.995 (0.01)
		NM	0.995 (0.01)	0.995 (0.01)	0.996 (0.01)	0.996 (0.01)	0.938 (0.04)	0.938 (0.04)
		Step-Down	0.479 (0.32)	0.479 (0.32)	0.458 (0.33)	0.458 (0.33)	0.994 (0.02)	0.994 (0.02)
		Min-Max	0.992 (0.01)	0.992 (0.01)	**0.997 (0.00)**	**0.997 (0.00)**	**0.997 (0.01)**	**0.997 (0.01)**
$M=3$, $N=(30,30,30)$	5	SCOR	**0.902 (0.09)**	**0.907 (0.07)**	**0.975 (0.07)**	**0.975 (0.07)**	**0.749 (0.06)**	**0.747 (0.06)**
		NM	0.880 (0.06)	0.877 (0.06)	0.961 (0.04)	0.960 (0.04)	0.523 (0.11)	0.528 (0.11)
		Step-Down	0.336 (0.35)	0.337 (0.35)	0.379 (0.38)	0.371 (0.38)	0.721 (0.08)	0.718 (0.09)
		Min-Max	0.770 (0.10)	0.772 (0.10)	0.906 (0.08)	0.907 (0.08)	0.646 (0.09)	0.651 (0.09)
	10	SCOR	**0.991 (0.01)**	**0.981 (0.10)**	**0.995 (0.01)**	**0.985 (0.10)**	**0.970 (0.03)**	**0.970 (0.03)**
		NM	0.963 (0.03)	0.961 (0.04)	0.984 (0.02)	0.983 (0.03)	0.707 (0.09)	0.715 (0.09)
		Step-Down	0.374 (0.36)	0.374 (0.36)	0.248 (0.31)	0.250 (0.31)	0.964 (0.03)	0.964 (0.03)
		Min-Max	0.942 (0.04)	0.942 (0.04)	0.968 (0.05)	0.968 (0.05)	0.909 (0.04)	0.909 (0.04)
	15	SCOR	**0.994 (0.01)**	**0.984 (0.10)**	**0.995 (0.01)**	**0.995 (0.01)**	**0.986 (0.02)**	**0.986 (0.02)**
		NM	0.975 (0.10)	0.958 (0.17)	0.990 (0.02)	0.973 (0.14)	0.798 (0.08)	0.798 (0.08)
		Step-Down	0.294 (0.37)	0.293 (0.37)	0.400 (0.36)	0.403 (0.36)	0.977 (0.05)	0.977 (0.05)
		Min-Max	0.966 (0.05)	0.966 (0.05)	0.989 (0.03)	0.989 (0.03)	0.962 (0.03)	0.962 (0.03)

Table S3: Performance comparison for two and three ordinal outcomes, where each class has sample size 60 for the two category case, and 30 for the three category case. The empirical hypervolume under manifolds (EHUM) and upper and lower bound approach (ULBA) objective functions are maximized by the proposed Spherically Constrained Optimization Routine (SCOR) algorithm and the existing Nelder-Mead (NM), step-down, and min-max algorithms. The estimated biomarker coefficient vectors are then used to calculate the EHUM value on a new dataset of the same size generated from the corresponding model. The entire procedure is repeated 100 times, resulting in 100 simulated training and test data sets, and the mean EHUM objective function values on the test data are reported, with the standard error in the parentheses. The result for the method with the best performance is marked in bold.

To show empirically how the mean squared error (MSE) of the estimated optimal coefficient vector $\boldsymbol{\beta}$ changes with sample size, we calculate the MSE of the estimated coefficient vector obtained using the SCOR algorithm maximizing both the ULBA and EHUM objective functions for simulation Scenario 1 (described in the simulation study section in the main paper) based on 100 simulation replications. Following Su & Liu (1993), it can be shown that if $\boldsymbol{X}_1, \boldsymbol{X}_2, \ldots, \boldsymbol{X}_M$ are multivariate normally distributed with mean vectors $\boldsymbol{\mu_1}, \ldots, \boldsymbol{\mu}_M$, respectively, and common variance-covariance matrix $\boldsymbol{\Sigma}$ satisfying

$$\boldsymbol{\mu}_2 - \boldsymbol{\mu}_1 = \boldsymbol{\mu}_3 - \boldsymbol{\mu}_2 = \cdots = \boldsymbol{\mu}_M - \boldsymbol{\mu}_{M-1} = \boldsymbol{\delta},$$

then the optimal coefficient parameters is proportional to $\boldsymbol{\Sigma}^{-1}\boldsymbol{\delta}$. Following this criteria, the true $\boldsymbol{\beta}$ (subject to the condition $||\boldsymbol{\beta}||_2 = 1$) is calculated for Scenario 1. The MSE is given by:

$$MSE = \frac{1}{n}\sum_{i=1}^{n} ||\boldsymbol{\beta} - \hat{\boldsymbol{\beta}}_i||_2^2$$

where $\boldsymbol{\beta}$ denotes the true coefficient vector and $\hat{\boldsymbol{\beta}}_i$ denotes the estimated coefficient vector at the i-th replication of the simulation study; $n = 100$. In Table S4 we note that the MSE of the estimated coefficient vector decreases as sample size increases.

Sample sizes	ULBA	EHUM
N = (30,30)	0.487	0.487
N = (60,60)	0.462	0.462
N = (120,120)	0.453	0.453

Table S4: The mean squared error (MSE) of the estimated coefficient vector for 2-category case under simulation Scenario 1 of the main paper, obtained for ULBA and EHUM using the SCOR algorithm for the cases with sample sizes $N = (30, 30), (60, 60), (120, 120)$.

G Application to Alzheimer's disease data

Alzheimer's is a form of dementia which causes problems with thinking, memory and behaviour. The greatest known risk factor for Alzheimer's is increasing age, and it is more prevalent in people 65 years old or older. In 2010, 4.7 million Americans over the age of 65 years had Alzheimer's, and researchers predict that by 2050, it will increase to 13.8 million (Hebert et al. 2013). Early detection of Alzheimer's is crucial to slow down the worsening of dementia symptoms and to improve quality of life. We consider a dataset (Luo & Xiong

2012) consisting of measures on 14 neuropsychometric markers for 118 individuals. This dataset is available in the R package `DiagTest3Grp`. These 118 individuals can be divided into three diagnostic categories: healthy, mild cognitive impairment (MCI), and Alzheimers disease (AD). We disregard data on 10 individuals with missing observations. Out of the remaining 108 patients, the number of subjects in the healthy, MCI, and AD groups are 44, 43, and 21 respectively. The 14 markers correspond to numeric measurements on the following neuropsychometric tests: global (*factor1*), temporal (*ktemp*), parietal (*kpar*), frontal (*kfront*), logical memory (*zpsy004*), digital span forward (*zpsy005*), digital span backward (*zpsy006*), information (*zinfo*), two measures of visual retention (*zbentc*, *zbentd*), Boston naming (*zboston*), mental control (*zmentcon*), word fluency (*zworflu*), and associate learning (*zassc*). Since *factor1*, *ktemp* and *zpsy004* are highly correlated (Luo & Xiong 2012), we only consider the *ktemp* biomarker out of these three markers, as it has the highest individual EHUM value. So, in total, 12 biomarkers are included in the analysis.

To illustrate the relative performance of the SCOR, step-down and min-max techniques, we compute the estimated combination coefficients maximizing the EHUM and ULBA criteria using each technique. Then EHUM value at those solutions are evaluated. We also compute the optimal cut-points to categorize the combination scores (obtained by multiplying the optimal coefficient vector with the biomarker values) using Youden's index (Youden 1950). Youden's index is defined as the maximum possible value of (sensitivity + specificity − 1) over all possible decision thresholds, and provides a summary measure combining sensitivity and specificity (Luo & Xiong 2012). Note that before obtaining the combination scores, all the solution combination vectors obtained in the step-down and min-max techniques are divided by their corresponding norms so that each solution vector has norm 1.

Figure S1 shows that using SCOR we obtain both higher values of EHUM and Youden's Index across all three objective functions (ULBA and EHUM). In addition, the cut-points obtained distinguish the three outcome categories more clearly for SCOR than step-down or min-max. In Table S5 we provide the values of the optimal combination coefficients using SCOR and step-down. Note that the signs of the estimated coefficients of the biomarkers are more consistent across the objective functions EHUM and ULBA for SCOR compared to the step-down approach. We obtain the highest value of $D_E(\hat{\beta})$ using the estimate obtained by maximizing EHUM/ULBA with the SCOR algorithm, the coefficients obtained using this approach (i.e., those given in the first column) represent the preferred solution.

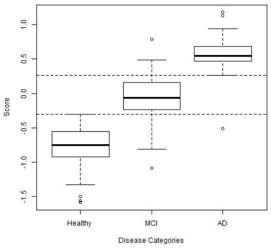

(a) EHUM (SCOR)
$D_E = 0.849, YI = \mathbf{0.802}$

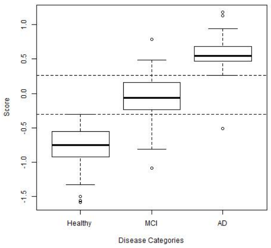

(b) ULBA (SCOR)
$D_E = 0.849, YI = \mathbf{0.802}$

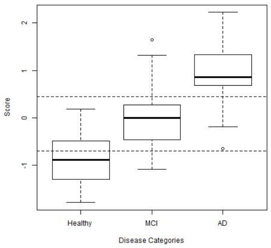

(c) EHUM (Step-down)
$D_E = 0.747, YI = 0.630$

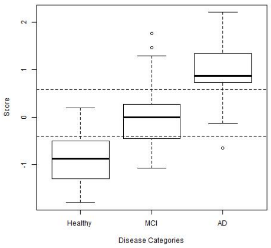

(d) ULBA (Step-down)
$D_E = 0.750, YI = 0.651$

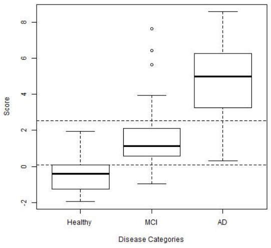

(e) EHUM (Min-max)
$D_E = 0.783, YI = 0.687$

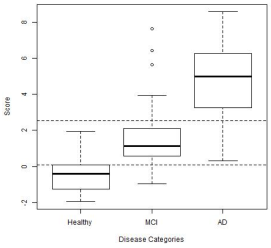

(f) ULBA (Min-max)
$D_E = 0.783, YI = 0.687$

Figure S1: Boxplots of the optimal combination vector scores are shown across the outcome categories healthy, MCI, and AD. The methods compared are SCOR ((a) EHUM, (b) ULBA), Step-down ((c) EHUM, (d) ULBA) and Min-max ((e) EHUM, (f) ULBA). The horizontal dotted lines denote the corresponding cut-points for classification obtained by maximizing Youden's Index.

Markers	ULBA (SCOR)	EHUM (SCOR)	ULBA (ST)	EHUM (ST)
ktemp	-0.360	-0.360	-0.6440	-0.622
kpar	-0.084	-0.084	0.1660	0.160
kfront	-0.367	-0.367	-0.2280	-0.221
zpsy005	0.206	0.206	-0.3470	-0.436
zpsy006	-0.204	-0.204	0.2230	0.215
zinfo	0.694	0.694	0	0
zbentc	-0.160	-0.160	0.1140	0.128
zbentd	0.251	0.251	-0.2230	-0.170
zboston	-0.047	-0.047	0.0950	0.091
zmentcon	0.228	0.228	0.2560	0.253
zworflu	0.108	0.108	0.0600	0.057
zassc	-0.095	-0.095	0.4410	0.426
$D_E(\hat{\beta})$	0.849	0.849	0.750	0.747

Table S5: Optimal coefficients obtained by maximizing the ULBA and EHUM objective functions using the SCOR and step-down algorithms. The EHUM objective function values at all the obtained solutions are reported in the last row.

References

Audet, C., Bechard, V. & Digabel, S. L. (2008), 'Nonsmooth optimization through mesh adaptive direct search and variable neighborhood search', *Journal of Global Optimization* **41**(2), 299–318.

Audet, C. & Dennis, J. (2006), 'Mesh adaptive direct search algorithms for constrained optimization', *SIAM Journal on Optimization* **17**(1), 188–217.

Bethke, A. D. (1980), 'Genetic algorithms as function optimizers'.

Conn, A., Scheinberg, K. & Vicente, L. (2009), 'Introduction to derivative-free optimization', *Mathematics without boundaries: Surveys in interdisciplinary research, MOS-SIAM Series on Optimization, SIAM* .

Digabel, S. L. (2011), 'Algorithm 909: Nomad: Nonlinear optimization with the mads algorithm', *ACM Transactions on Mathematical Software* **37**(4(44)), 1–15.

Fraser, A. (1957), 'Simulation of genetic systems by automatic digital computers i. introduction', *Australian Journal of Biological Sciences* **10**, 484–491.

Geris, L. (2012), *Computational Modeling in Tissue Engineering*, Springer.

Granville, V., Krivanek, M. & Rasson, J. P. (1994), 'Simulated annealing: A proof of convergence', *IEEE Transactions on Pattern Analysis and Machine Intelligence* **16**, 652–656.

Hebert, L., Weuve, J., Scherr, P. & Evans, D. (2013), 'Alzheimer disease in the united states (20102050) estimated using the 2010 census', *Neurology* **80**(19), 1778–1783.

Hooke, R. & Jeeves, T. A. (1961), 'Direct search solution of numerical and statistical problems', *Journal of the Association for Computing Machinery* **8**, 212–219.

Hsu, M. & Chen, Y. (2016), 'Optimal linear combination of biomarkers for multi-category diagnosis', *Statistics in Medicine* **35**(2), 202–213.

Jamil, M. & Yang, X. (2013), 'A literature survey of benchmark functions for global optimization problems', *Int. J. of Mathematical Modelling and Numerical Optimisation* **4**(2).

Karmakar, N. (1984), 'New polynomial-time algorithm for linear programming', *Combinatorica* **4**, 373–395.

Kirkpatrick, S., Gelatt, C. & Vecchi, M. (1983), 'Optimization by simulated annealing', *Australian Journal of Biological Sciences* **220**(4598), 671–680.

Kolda, T., Lewis, R. & Torczon, V. (2003), 'Optimization by direct search: New perspectives on some classical and modern methods', *SIAM Review* **45**(3), 385–482.

Liu, C., Liu, A. & Halabi, S. (2011), 'A minmax combination of biomarkers to improve diagnostic accuracy', *Statistics in Medicine* **30**(16), 2005–2014.

Luo, J. & Xiong, C. (2012), 'Diagtest3grp: An r package for analyzing diagnostic tests with three ordinal groups', *J Stat Softw.* **51**(3), 1–24.

Maiti, R., Li, J., Das, P., Feng, L., Hausenloy, D. & Chakraborty, B. (2019), 'A distribution-free smoothed combination method of biomarkers to improve diagnostic accuracy in multi-category classification', *arxiv.org/abs/1904.10046* .

Nelder, J. & Mead, R. (1965), 'A simplex method for function minimization', *Computer Journal* **7**, 308–313.

Pepe, M., Cai, T. & Longton, G. (2006), 'Combining predictors for classification using the area under the receiver operating characteristic curve', *Biometrics* **62**(1), 221–229.

Potra, F. & Wright, S. (2000), 'Interior-point methods', *Journal of Computational and Applied Mathematics* **4**, 281–302.

P.T. Boggs, J. T. (1996), 'Sequential quadratic programmings', *Acta Numerica* pp. 1–52.

Steihaug, T. & Suleiman, S. (2013), 'Global convergence and the powell singular function', *Journal of Global Optimization* **56**(3), 845–853.

Su, J. & Liu, J. (1993), 'Linear combinations of multiple diagnostic markers', *Journal of the American Statistical Association* **88**(424), 1350–1355.

Torczon, V. (1997), 'On the convergence of pattern search algorithms', *SIAM Journal on Optimization* **7**, 1–25.

Wright, M. (2005), 'The interior-point revolution in optimization: History, recent developments, and lasting consequences', *Bulletin of American Mathematical Society* **42**, 39–56.

Youden, W. (1950), 'Index for rating diagnostic tests', *Cancer* **3**, 32–35.
